# Comprehensive Transcriptomic Analysis Identifies Potential Biomarkers and Underlying Mechanisms of Acupuncture in Treating Peripheral Facial Paralysis

**DOI:** 10.1007/s12031-026-02599-6

**Published:** 2026-10-10

**Authors:** Xihong Li, Yan Li, Xinyu Zou, Juanjuan Liu, Runyu Yin, Qinglin Wei, Yonghua Hu

**Affiliations:** 1 The Second Affiliated Hospital (Affiliated Hospital of Gansu, University of Chinese Medicine), Gansu University of Chinese Medicine, Lanzhou, 730000 China; 2 First School of Clinical Medicine, Gansu University of Chinese Medicine, Lanzhou, 730000 China; 3 Affiliated Hospital of Gansu University of Chinese Medicine (North Branch), Lanzhou, 730000 China; 4 First Teaching Hospital of Tianjin University of Traditional Chinese Medicine, National Clinical Research Center for Chinese Medicine Acupuncture and Moxibustion, Tianjin, 300381 China

## Abstract

Peripheral facial palsy (PFP) is a common neurological disorder. Acupuncture is an effective therapeutic intervention, yet its underlying molecular mechanisms remain incompletely elucidated. To evaluate the clinical efficacy of acupuncture for PFP and to identify differentially expressed RNAs and associated pathways via whole transcriptome analysis, thereby revealing potential molecular mechanisms. Clinical efficacy was assessed using the House-Brackmann (HB) scale, Facial Disability Index (FDI), and comprehensive traditional Chinese medicine efficacy criteria. Peripheral blood samples were collected from patients before and after acupuncture treatment. Differentially expressed circRNAs, lncRNAs, miRNAs, and mRNAs were screened through whole transcriptome sequencing. Bioinformatics analyses were employed to construct a competing endogenous RNA (ceRNA) network and perform Gene Ontology (GO) and Kyoto Encyclopedia of Genes and Genomes (KEGG) pathway enrichment analyses. Acupuncture significantly improved clinical scores post-treatment. A total of 1625 differentially expressed mRNAs (1153 up-, 472 down-regulated), 715 lncRNAs (321 up-, 394 down-regulated), 85 circRNAs (56 up-, 29 down-regulated), and 253 miRNAs (141 up-, 112 down-regulated) were identified. The ceRNA network revealed that non-coding RNAs competitively bind key miRNA molecules, forming two distinct and independent regulatory axes that precisely modulate downstream target genes. KEGG enrichment analysis of core molecular targets indicated significant involvement in the Olfactory transduction and JAK-STAT signaling pathways. Acupuncture may exert its therapeutic effect on PFP by modulating the ceRNA network, alongside the Olfactory transduction and JAK-STAT signaling pathways, synergistically promoting nerve regeneration and suppressing neuroinflammation.

## Introduction

Facial palsy, resulting from injury to the lower motor neurons of the facial nerve, is a prevalent clinical condition. It primarily manifests as reduced forehead wrinkles, shallow or absent nasolabial fold on the affected side, and may be accompanied by incomplete eyelid closure, taste disturbance, otalgia, and facial numbness(Zhou et al. 2022, Cheng et al. 2023). The facial nerve, a mixed nerve controlling facial muscle movement and gland secretion, is the most susceptible cranial nerve to paralysis(Soloperto et al. 2020). Peripheral facial palsy (PFP) represents the most common form encountered clinically (Lassaletta et al. 2020, Pons et al. 2013). The incidence of PFP ranges from 20 to 30 per 100,000 individuals, with approximately 40,000 new cases annually worldwide and no significant gender predominance(Fu et al. 2018). Unilateral facial muscle paralysis often leads to psychological issues such as anxiety and depression, resulting in social impairment and diminished quality of life(Cuenca-Martínez et al. 2020).

A 2020 meta-analysis on facial paralysis suggested that the efficacy of steroids combined with antiviral therapy for acute facial palsy remains controversial(Kim and Lee 2020). In China, acupuncture is a commonly employed clinical treatment. Existing clinical studies have demonstrated the effectiveness of acupuncture for PFP(Yu et al. 2023). Furthermore, network meta-analysis has been conducted to rank the efficacy of different acupuncture methods(Li et al. 2020).

Acupuncture exhibits significant advantages in PFP treatment—it is straightforward to perform, demonstrates notable efficacy(Li and Liu 2005), possesses a favorable safety profile, and is cost-effective. However, the mechanism of action of acupuncture for PFP is not fully understood. While previous research has explored pathways such as nerve repair, immune regulation, and improvement of local microcirculation(Duan et al. 2025), a systematic analysis of the “gene-pathway-efficacy” relationship at the transcriptome level is lacking. Therefore, this self-controlled clinical trial aimed to evaluate the clinical efficacy and safety of acupuncture in PFP patients. From a transcriptomic perspective, we utilized high-throughput sequencing to screen differentially expressed genes in peripheral blood of PFP patients following acupuncture intervention. Subsequent bioinformatics analysis was employed to identify key signaling pathways enriched with these differential genes, thereby constructing a “gene-pathway-efficacy” correlation network.

## Materials and Methods

### Study Subjects

This study enrolled PFP patients from the Acupuncture Center and North Branch Acupuncture Department of the Affiliated Hospital of Gansu University of Chinese Medicine between April 2024 and December 2024. All participants provided written informed consent voluntarily.

Inclusion criteria were: ① Meeting the diagnostic criteria for idiopathic facial nerve palsy according to the 9th edition of “Neurology” published by People’s Medical Publishing House, with a disease course exceeding three months; ② Age between 18 and 80 years, regardless of gender; ③ No relevant treatment received within the preceding 2 weeks; ④ Signed informed consent and ability to actively cooperate with the treatment protocol. All patients received standardized acupuncture treatment without a separate control group, utilizing a pre-post self-paired design.

### Acupuncture Treatment

Patients assumed a supine position with the treatment areas adequately exposed. Following routine local disinfection: bilateral Hegu (LI4) were perpendicularly needled to a depth of 0.8 cun, applying lifting-thrusting reinforcing technique; affected-side Sibai (ST2), Quanliao (SI18), Yifeng (TE17), Taiyang (EX-HN5), and Xiaguan (ST7) were perpendicularly needled to 0.5 cun; Yangbai (GB14) on the affected side was needled subcutaneously to 0.5 cun using the pinching-up method; Jiache (ST6) on the affected side was obliquely needled towards Dicang (ST4) to 0.5 cun, and Dicang (ST4) was obliquely needled towards Jiache (ST6) to 0.5 cun. Even reinforcing-reducing technique was applied to the aforementioned points, and needles were retained for 30 min. Treatments were administered every other day, with 5 sessions constituting one course, totaling 3 courses.

### Efficacy and Safety Evaluation

Efficacy evaluation criteria referenced the House - Brackmann Facial Grading System(H-B)(House and Brackmann 1985), Facial Disability Index scale (FDI) (VanSwearingen and Brach 1996), comprehensive Traditional Chinese Medicine (TCM) symptom efficacy standards for facial palsy, and safety assessments. Facial muscle strength and symmetry of forehead wrinkling/eye closure were observed. The House-Brackmann (H-B) scale was used to grade facial nerve function into levels I, II, III, IV, V, and VI, scored as 5, 4, 3, 2, 1, and 0 points respectively, with higher scores indicating milder symptoms. The FDI comprises the Physical Function (FDIP) and Social/Well-being Function (FDIS) subscales. FDIP assesses difficulty with eating, drinking, speaking, tearing, and rinsing mouth; higher scores indicate better physical function. FDIS assesses feelings of calmness, isolation, anger, sleep, and social interaction duration; lower scores indicate better social life ability. The comprehensive TCM symptom efficacy evaluation standard was formulated with reference to the “Guiding Principles for Clinical Research of New Chinese Medicines” (2002 trial version) (State Administration of Traditional Chinese Medicine 1994), utilizing TCM syndrome scores to evaluate overall clinical efficacy. Adverse events such as needle fainting, stuck needle, broken needle, or post-needling hematoma were monitored. The occurrence time, severity, countermeasures taken, and effectiveness of measures were recorded. Treatment could be suspended or participants could withdraw from the study if necessary.

### Transcriptome Sequencing and Analysis Pipeline

#### RNA Quantification and Library Construction

RNA degradation and contamination, particularly DNA contamination, were monitored on 1.5% agarose gels. RNA concentration and purity were measured using advanced molecular biology equipment to ensure sample qualification for transcriptome sequencing. Nanodrop was used to assess RNA purity (OD260/280 ≥ 1.8, OD260/230 ≥ 0.5, normal peak at 260 nm) and concentration (≥ 50 ng/µl). Agilent 2100 Bioanalyzer precisely determined RNA integrity (RIN ≥ 7.0; 28 S/18S ≥ 1.5; baseline without elevation; normal 5 S peak). Main library construction steps: (1) Enrichment of eukaryotic mRNA using magnetic beads with Oligo; (2) Fragmentation of mRNA using Fragmentation Buffer; (3) Synthesis of first-strand cDNA using mRNA as template and random hexamers, followed by synthesis of second-strand cDNA using buffer, dNTPs, RNase H, and DNA polymerase I, purification with AM Pure XP beads; (4) End repair, A-tailing, and adapter ligation performed on purified double-stranded cDNA, followed by fragment size selection with AM Pure XP beads; (5) PCR enrichment to obtain the final cDNA library. Post-construction, library quality was inspected. Libraries meeting requirements proceeded to sequencing. Qubit 2.0 was used for preliminary quantification. Agilent 2100 was used to detect insert size, proceeding only if insert size met expectations. Q-PCR accurately quantified the effective library concentration (effective concentration > 2 nM) to complete library inspection.

#### Raw Data Processing

FastQC software assessed the quality of raw sequencing data. Trimmomatic software filtered out low-quality reads (Q20 < 90%), adapter sequences, and contaminant sequences, retaining high-quality clean reads. Clean reads were aligned to the human reference genome (hg38). HISAT2 software aligned mRNA reads, and Bowtie software aligned miRNA reads, with alignment efficiency required to be ≥ 80%.

### Screening of Differential RNAs Pre- and Post-Treatment

A paired-sample differential analysis strategy was employed. DESeq2 software performed differential expression analysis on serum samples from 8 patients collected before and after treatment. Specific screening thresholds were set to identify differentially expressed mRNAs, lncRNAs, miRNAs, and circRNAs. Volcano plots visualized the distribution of differential molecules, and heatmaps displayed the expression clustering patterns of core differential molecules. Different screening criteria applied to different RNA types (See Supplementary Material).

### Functional Annotation and Pathway Enrichment Analysis

Differentially expressed molecules were subjected to Gene Ontology (GO) functional enrichment analysis and KEGG pathway enrichment analysis using the Metascape database. GO analysis covered Biological Process (BP), Cellular Component (CC), and Molecular Function (MF) categories. KEGG analysis focused on signal pathway enrichment. A P-value < 0.05 was considered statistically significant. ClusterProfiler (v4.4.4) was used to visualize GO functional bar plots and KEGG pathway bubble plots.

### ceRNA Network Construction

Competitive endogenous RNAs (ceRNAs) represent a recent research focus and a novel transcriptional regulatory model (Salmena et al. 2011). ceRNAs can mutually regulate their expression levels by competitively binding the same miRNA via miRNA response elements. The ceRNA hypothesis reveals a new mechanism of RNA interaction. mRNAs, lncRNAs, and circRNAs can all function as ceRNAs. RNAhybrid v2.1.1, miRanda v3.3a, and TargetScan v5.0 were used jointly to predict targeting relationships, supplemented by TargetFinder v1.6 for validation to reduce false positives from single algorithms. Targeting prediction for circRNAs was based on their own sequences (sourced from the CircInteractome database), not substituted by their host gene targeting relationships. Based on prediction results, core differential molecules for different RNAs were screened. Venny 2.1 software generated Venn diagrams for visualization. Following the core ceRNA regulatory rule, complete regulatory chains conforming to “lncRNA/circRNA and mRNA showing consistent expression trends, miRNA showing opposite expression trend to both” were further screened. Finalized targeting relationships were formatted as “source node - target node” and imported into Cytoscape 3.9.1 software to construct and visualize the ceRNA network, clarifying nodes and edges. KEGG enrichment analysis was performed on the screened core molecules to identify the specific pathways they drive, visualized using bar plots.

### Statistical Analysis

This study adopted a self-controlled paired design, in which FDI facial disability index scores and House–Brackmann (H-B) facial nerve grading data were collected from all subjects before and after treatment. All experimental data were statistically analyzed using SPSS 26.0. The normality test was performed on the differences between pre-treatment and post-treatment scores. If the differences followed a normal distribution, paired-samples t test was used; otherwise, the Wilcoxon signed-rank test was applied to assess intra-group differences. The median difference and its 95% confidence interval were calculated via the Hodges–Lehmann estimator, and corresponding effect sizes were also computed. A P value < 0.05 was considered statistically significant.

Pathway enrichment analysis and screening of RNA-target regulatory relationships were performed based on differentially expressed genes. Raw P values obtained from all tests were adjusted for multiple hypothesis testing using the Benjamini–Hochberg (BH) method. The adjusted FDR was defined as the q value; a smaller q value indicated higher statistical reliability of the enrichment or target association. An identical correction algorithm and significance criterion were uniformly applied to all RNA species (mRNA, lncRNA, circRNA and miRNA) in this study. Only adjusted q values (FDR) rather than raw P values were used to determine statistical significance, with the screening threshold set at FDR < 0.05.

## Results

### Efficacy and Safety Evaluation Results

#### Facial Nerve Function Score (H-B Scale) Results

H-B scale results are categorical data, presented as n (%). Chi-square test or Fisher’s exact test showed a statistically significant difference in H-B scores before and after treatment (*P* < 0.001). Details are in Table 1.

**Table 1:** Comparison of Facial Nerve Function Scores Before and After Treatment

H-B, *n*(%)	Total (*n* = 16)	Pre-Treatment (*n* = 8)	Post-Treatment (*n* = 8)	Statistic	*P*	
				-	< 0.001	
I	5 (31.25)	0 (0.00)	5 (62.50)			
Ⅱ	3 (18.75)	0 (0.00)	3 (37.50)			
Ⅲ	1 (6.25)	1 (12.50)	0 (0.00)			
Ⅳ	2 (12.50)	2 (25.00)	0 (0.00)			
Ⅴ	4 (25.00)	4 (50.00)	0 (0.00)			
Ⅵ	1 (6.25)	1 (12.50)	0 (0.00)			

-: Fisher exact

#### Physical Function (FDIP) Score Results

Normality test revealed that the differences in FDIP scores between pre-treatment and post-treatment did not follow a normal distribution; thus, the Wilcoxon signed-rank test was used for self-paired comparisons. The median pre-treatment FDIP score was 40.00 (30.00, 57.50), which increased to 100.00 (77.50, 100.00) after treatment. The median paired difference was 46.25 (37.5, 57.5). The Wilcoxon test yielded Z = -2.527, *P* = 0.012, indicating a statistically significant improvement in physical function following treatment. Details are in Table 2.

**Table 2:** Comparison of physical function (FDIP) and social function (FDIS) scores in patients before and after treatment

Evaluation indicator	Pre-treatment Mdn (IQR)	Post-treatment Mdn (IQR)	Hodges–Lehmann median difference (95% CI)	Z	*P*
FDIP	40.00 (30.00, 57.50)	100.00 (77.50, 100.00)	46.25 (37.5, 57.5)	-2.527	0.012
FDIS	64.00 (61.00, 70.00)	28.00 (12.00, 36.00)	-40.00 (-48.00, -30.00)	-2.524	0.012

Mdn = median; IQR = interquartile range. The median paired difference was estimated using the Hodges-Lehmann estimator. As score differences were non-normally distributed, the Wilcoxon matched-pairs signed-rank test was performed

#### Social Function (FDIS) Score Results

Normality test indicated that the differences in FDIS scores before and after treatment were not normally distributed; therefore, the Wilcoxon signed-rank test was adopted for self-paired comparisons. The median pre-treatment FDIS score was 64.00 (61.00, 70.00), which decreased to 28.00 (12.00, 36.00) after treatment. The median paired difference was − 40.00 (-48.00, -30.00). The Wilcoxon test produced Z = -2.524, *P* = 0.012, suggesting a statistically significant improvement in social function after treatment.Details are in Table 2.

#### Comprehensive Efficacy Evaluation and Safety

After acupuncture treatment, the clinical overall efficacy index for all PFP patients exceeded 70%, reaching the “marked improvement” level or above. No adverse events occurred in any patient.

### Sample RNA Quality and Sequencing Data

RNA sample quality results: For mRNA, lncRNA, circRNA: Clean reads ratio ≥ 90%, Q30 base percentage (error rate ≤ 0.1%) ≥ 90%, GC content between 41% and 52%, unique mapping rate to reference genome (hg38) > 90%. For miRNA: Clean reads ratio ≥ 92%, Q30 base percentage ≥ 95%, alignment rate to human mature miRNA sequences in miRBase v22.0 database ranged from 68.64% to 91.05%.

### Differential RNA Screening

**Fig. 1:**
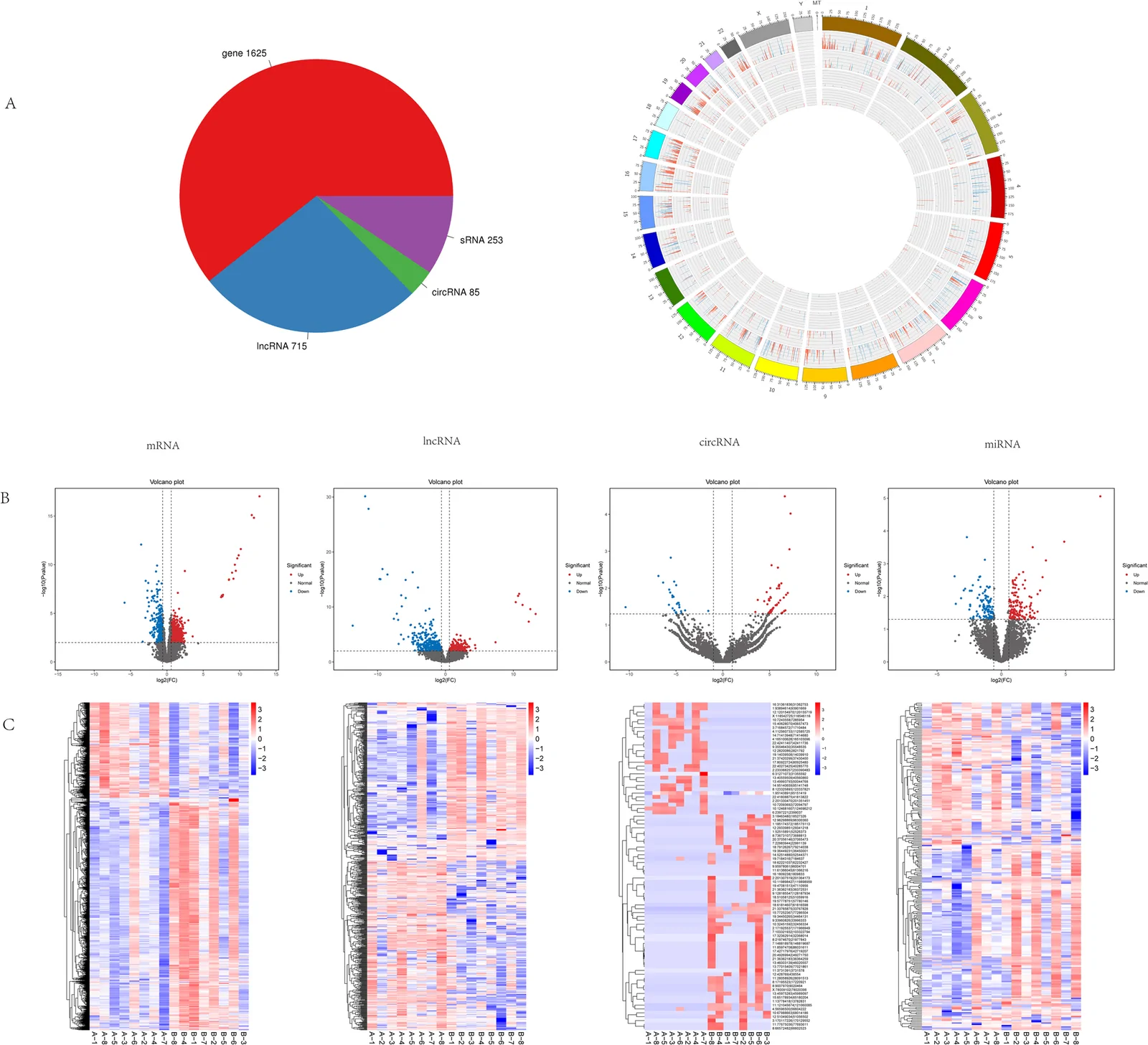
RNA Differential Screening Plots and Cluster Heatmap (**A**) Pie chart of differentially expressed RNA counts and global Circos plot; (**B**) Volcano plots of differentially expressed RNAs; (**C**) Cluster heatmap of differentially expressed RNAs

Via whole transcriptome high-throughput sequencing and bioinformatics analysis, this study systematically screened differentially expressed RNA molecules associated with acupuncture treatment for PFP. Applying specific thresholds, 1625 differentially expressed mRNAs (1153 up-, 472 down-regulated), 715 differentially expressed lncRNAs (321 up-, 394 down-regulated), 85 differentially expressed circRNAs (56 up-, 29 down-regulated), and 253 differentially expressed miRNAs (141 up-, 112 down-regulated) were identified (Fig. 1A, B). Cluster analysis of all differentially expressed RNAs (Fig. 1C) showed clear grouping clustering characteristics in the RNA expression profiles between pre- and post-treatment samples, indicating significant differences in RNA expression patterns.

### 3.4 Functional Annotation and Pathway Enrichment Analysis

**Fig. 2:**
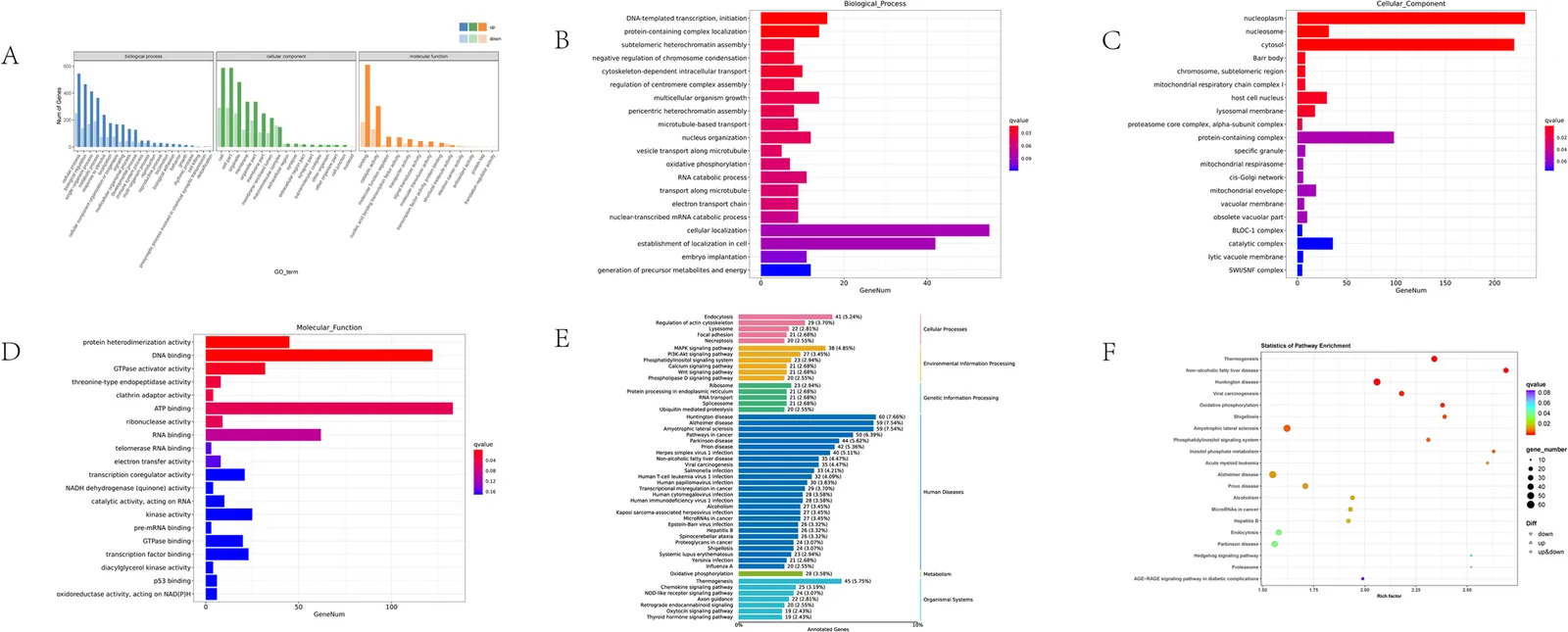
mRNA Functional Annotation and Enrichment Analysis (**A**)GO annotation classification bar plot; (**B**, **C**, **D**) GO enrichment bar plots for Biological Process, Molecular Function, Cellular Component respectively; (**E**) KEGG classification annotation plot; (**F**) KEGG enrichment bar plot

**Fig. 3:**
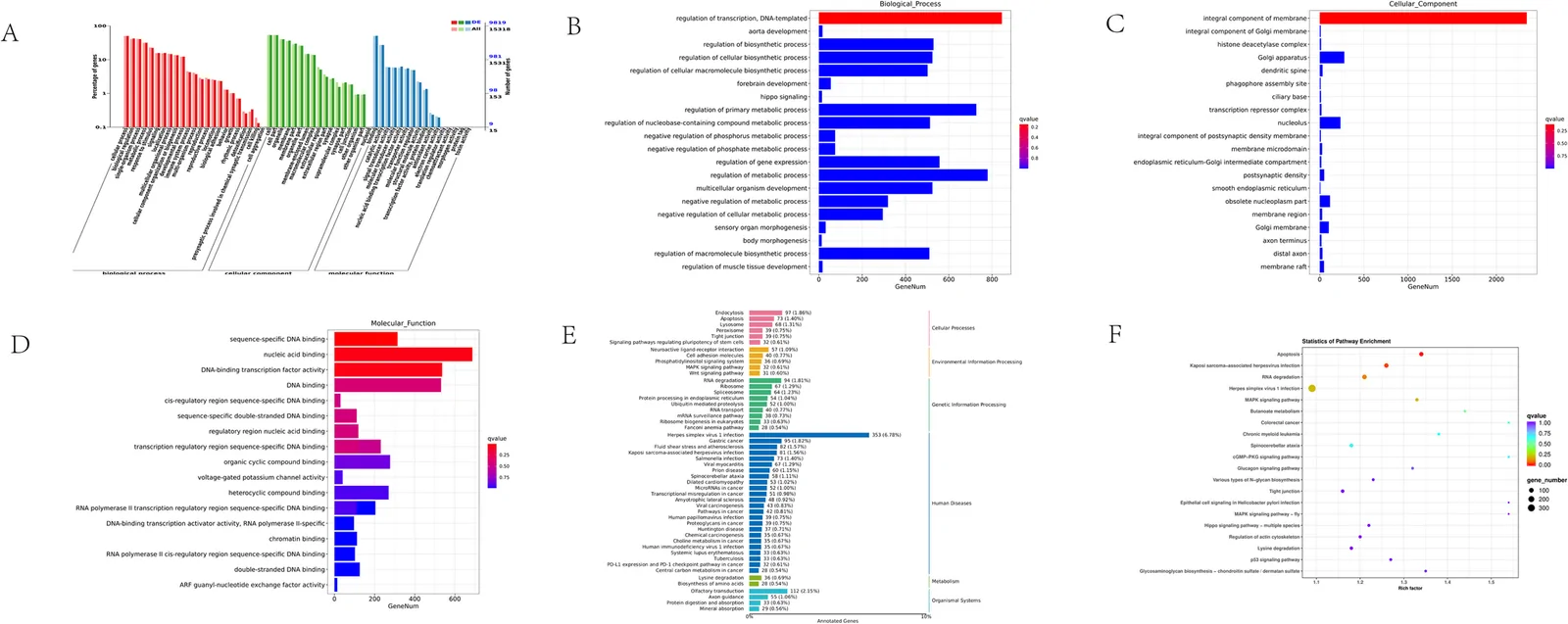
miRNA Functional Annotation and Enrichment Analysis (**A**)GO annotation classification bar plot; (**B**, **C**, **D**) GO enrichment bar plots for Biological Process, Molecular Function, Cellular Component respectively; (**E**) KEGG classification annotation plot; (**F**) KEGG enrichment bar plot

**Fig. 4:**
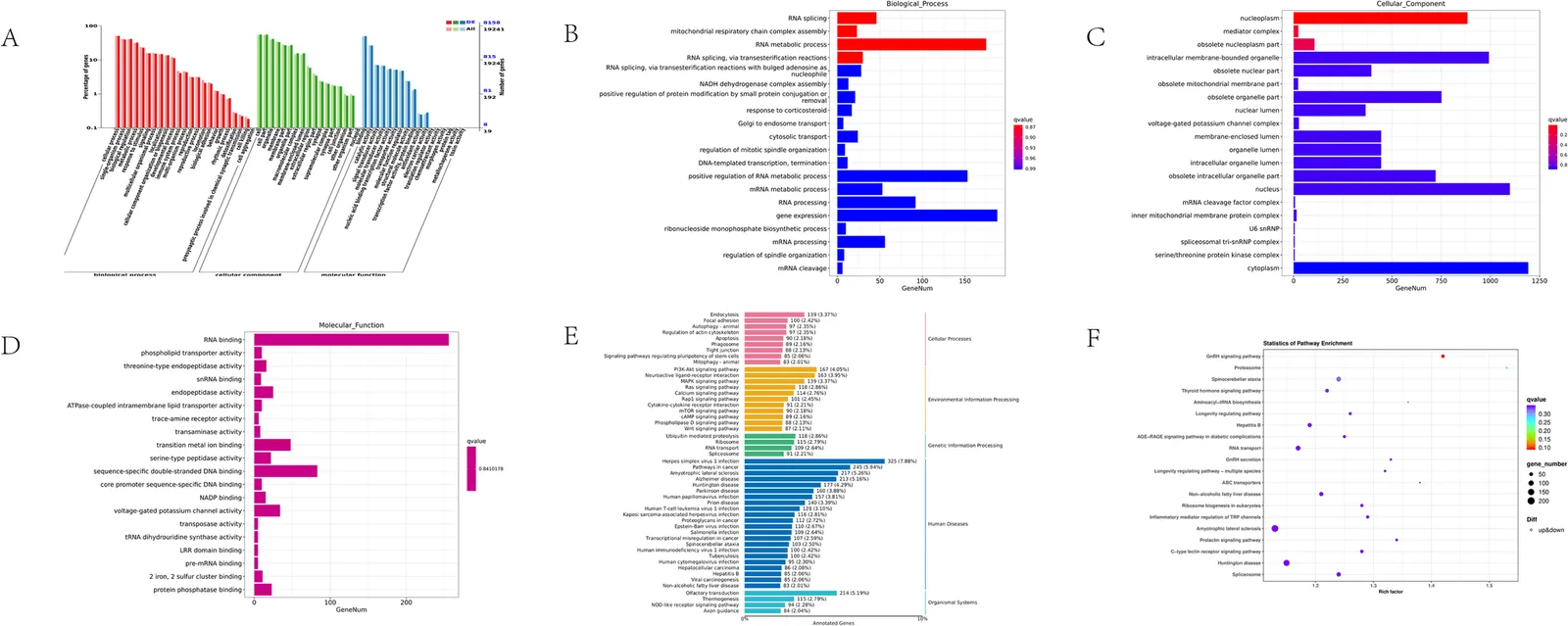
lncRNA Functional Annotation and Enrichment Analysis (**A**)GO annotation classification bar plot; (**B**, **C**, **D**) GO enrichment bar plots for Biological Process, Molecular Function, Cellular Component respectively; (**E**) KEGG classification annotation plot; (**F**) KEGG enrichment bar plot

**Fig. 5:**
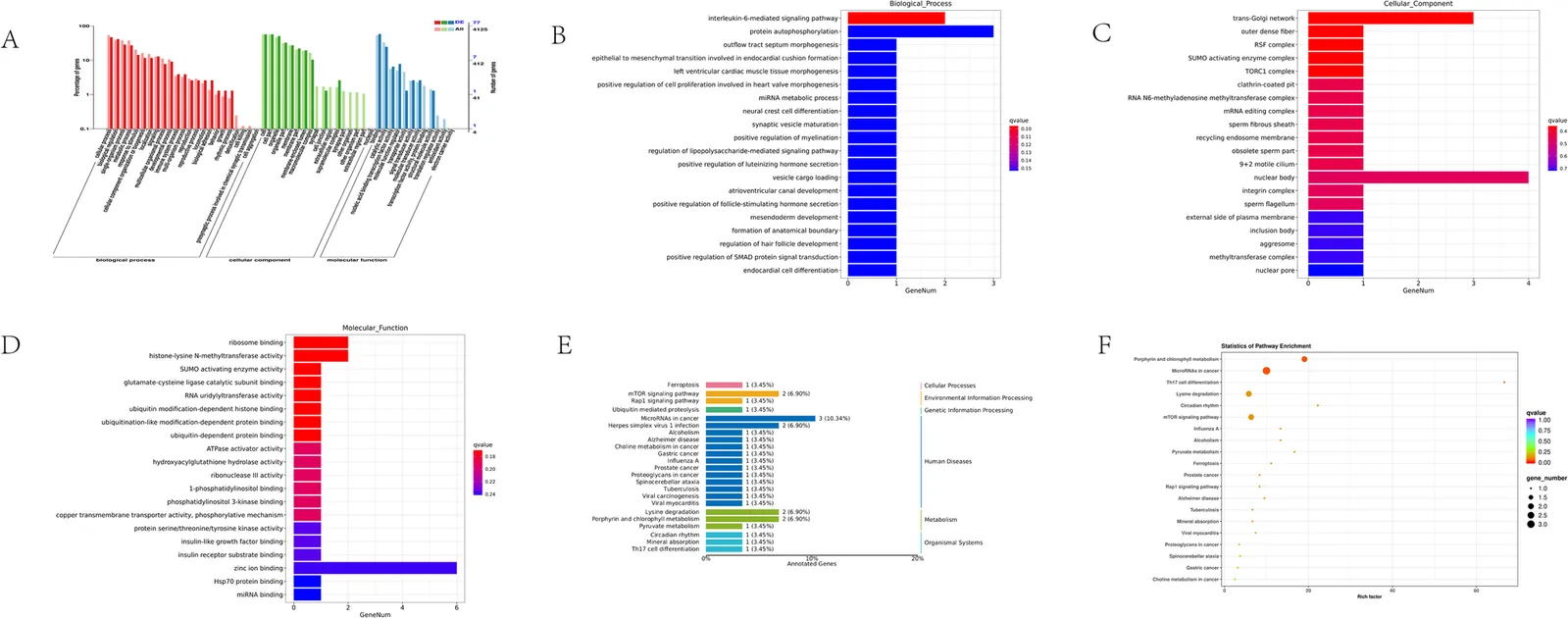
circRNA Functional Annotation and Enrichment Analysis (**A**) GO annotation classification bar plot; (**B**, **C**, **D**) GO enrichment bar plots for Biological Process, Molecular Function, Cellular Component respectively; (**E**) KEGG classification annotation plot; (**F**) KEGG enrichment bar plot

GO functional annotation results for differentially expressed mRNAs, miRNAs, lncRNAs, and circRNAs showed significant categorical tendencies (Figs. 2, 3, 4 and 5A). Biological Process (BP): Differential mRNAs were primarily enriched in cellular process, single-organism process, and biological regulation, with significant enrichment in cellular localization, establishment of intracellular localization, etc(Fig. 2B). miRNA core enrichment involved regulation of DNA-templated transcription, regulation of metabolic process, and regulation of primary metabolic process(Fig. 3B). lncRNAs concentrated on gene expression, RNA metabolic process, and positive regulation of RNA metabolic process(Fig. 4B). circRNAs were significantly enriched in interleukin-6-mediated signaling pathway and protein autophagic process(Fig. 5B). Cellular Component (CC): Differential mRNAs mainly belonged to cell, cell part, and organelle categories, with significant enrichment in nucleoplasm, cytosol, etc(Fig. 2C). miRNAs primarily localized to integral component of membrane(Fig. 3C). lncRNAs concentrated in nucleoplasm, cytoplasm, and nucleus(Fig. 4C). circRNAs enriched in trans-Golgi network and nuclear body(Fig. 5C). Molecular Function (MF): Differential mRNAs centered on binding and catalytic activity, with significant enrichment in ATP binding, DNA binding, etc(Fig. 2D). miRNAs mainly involved sequence-specific DNA binding, nucleic acid binding, and DNA-binding transcription factor activity(Fig. 3D). lncRNAs primarily functioned in RNA binding(Fig. 4D). circRNAs enriched in ribosomal binding, histone-lysine N-methyltransferase activity, and zinc ion binding(Fig. 5D).

KEGG annotation results for differential molecules predominantly featured human disease-related pathways(Figs. 2, 3, 4 and 5E), but enrichment characteristics varied: Differential mRNAs: Pathway with highest annotated gene ratio was Huntington disease, significantly enriched in Thermogenesis, Non-alcoholic fatty liver disease, and Huntington disease(Fig. 2F).Differential miRNAs: Pathway with highest annotated gene ratio was Herpes simplex virus 1 infection, significantly enriched in Apoptosis, Kaposi sarcoma-associated herpesvirus infection, and Herpes simplex virus 1 infection(Fig. 3F). Differential lncRNAs: Pathway with highest annotated gene ratio was also Herpes simplex virus 1 infection, significantly enriched in GnRH signaling pathway, Amyotrophic lateral sclerosis, and Huntington disease(Fig. 4F). Differential circRNAs: Pathway with highest annotated gene ratio was MicroRNAs in cancer, significantly enriched in Porphyrin and chlorophyll metabolism and MicroRNAs in cancer(Fig. 5F).

### ceRNA Network Construction Results

**Fig. 6:**
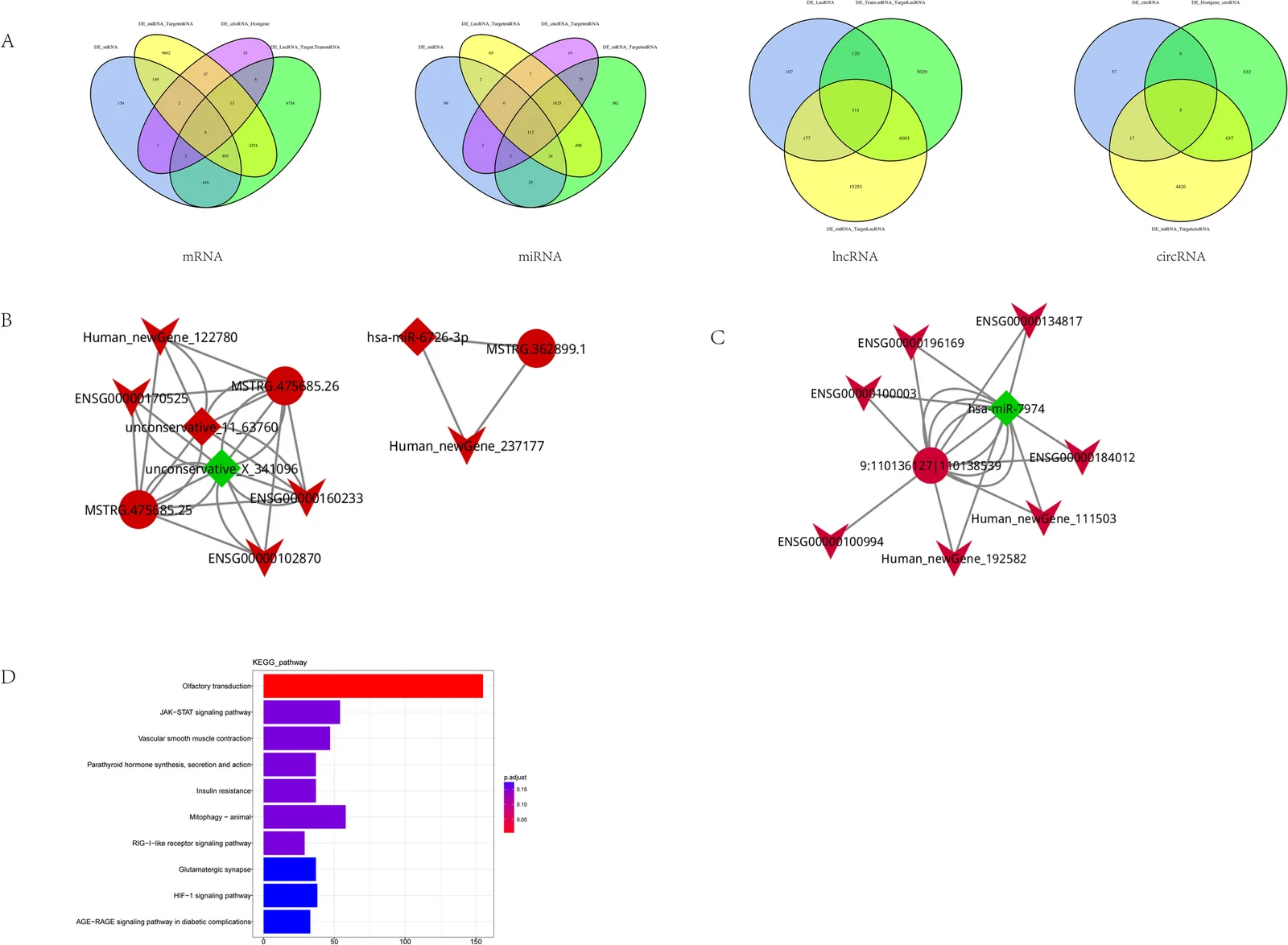
ceRNA Network Construction and Core Molecule Enrichment Pathways (**A**)Venn diagram of core molecules; (**B**) Differential lncRNA-miRNA-mRNA relationship plot; (**C**) Differential circRNA-miRNA-mRNA relationship plot; (**D**) KEGG enrichment bar plot for core molecules

Venn diagram analysis (Fig. 6A) indicated the numbers of core regulatory mRNAs, miRNAs, lncRNAs, and circRNAs were 6, 112, 311, and 5, respectively. The subsequently constructed ceRNA network comprised 20 nodes and 29 interaction edges (Fig. 6B, C), forming a synergistic “lncRNA/circRNA-miRNA-mRNA” regulatory model. Core regulatory nodes included: lncRNA nodes: MSTRG.475685.26, MSTRG.475685.25,MSTRG.362899.1.circRNAnode:9:110136127|110,138,539.miRNA nodes: hsa-miR-7974, unconservativeX_341096, unconservative_11_63760, hsa-miR-6726-3p.mRNAnodes: ENSG00000170525,ENSG00000160233, ENSG00000102870,Human_newGene_122780,Human_newGene_237177,ENSG00000134817,ENSG00000196169,ENSG00000100003,ENSG00000184012,ENSG00000100994, Human_newGene_111503, Human_newGene_192582.

Within the constructed differential ceRNA regulatory network, non-coding RNAs competitively bind key miRNA molecules, forming two independent and distinct regulatory pathways for precise modulation of downstream target genes: (1) circRNA (9:110136127|110138539) competitively binds the core miRNA (hsa-miR-7974), relieving its post-transcriptional repression of downstream target genes, thereby regulating the expression of known genes ENSG00000134817 (OR51E2), ENSG00000196169 (SOCS3), ENSG00000100003 (GABRA4), ENSG00000184012 (EGFR), ENSG00000100994 (NCAM1), and novel genes Human_newGene_111503 and Human_newGene_192582. (2) lncRNAs (MSTRG.475685.26 and MSTRG.475685.25), potentially via upregulating lncRNA unconservative_11_63760, synergistically competitively bind the core miRNA unconservativeX_341096, relieving its post-transcriptional repression of downstream known genes ENSG00000170525 (SEMA3A), ENSG00000160233 (PLXNB1), ENSG00000102870 (ROBO1), and novel gene Human_newGene_122780. Simultaneously, lncRNA (MSTRG.362899.1) potentially via upregulating miRNA (hsa-miR-6726-3p), targets and regulates the expression level of novel gene Human_newGene_237177.

KEGG pathway analysis of the screened core molecules revealed significant enrichment in the Olfactory transduction and JAK-STAT signaling pathways (Fig. 6D).

## Discussion

This study found that acupuncture treatment resulted in a clinical efficacy index exceeding 70% for PFP patients and significantly improved scale scores. Further integrated whole transcriptome analysis of patient serum samples revealed that acupuncture regulates target genes including OR51E2, SOCS3, GABRA4, EGFR, NCAM1, SEMA3A, PLXNB1, ROBO1, and several novel genes via the ceRNA network. KEGG enrichment analysis indicated that molecules within the ceRNA network are highly associated with the Olfactory transduction and JAK-STAT signaling pathways.

Peripheral facial palsy involves sensory and motor dysfunction following facial nerve injury(Chen et al. 2023). Facial nerve injury indirectly interrupts the olfactory transduction pathway via the trigeminal nerve (Li et al. 2009, Tremblay and Frasnelli 2018). The stimulatory properties of acupuncture align well with non-coding RNA axis activation—as a multi-target, gentle, and sustained physical stimulus, acupuncture can modulate the expression profile of circRNAs in plasma exosomes (Shou et al. 2019). The closed-loop structure of circRNAs confers greater stability than linear RNAs, allowing persistent presence in target tissues where they function as endogenous molecular sponges, specifically sequestering hsa-miR-7974 to maintain expression levels of downstream target genes. This “sustained signal regulation“(Moura et al. 2025) is particularly important for sensory functional recovery in the olfactory transduction pathway, suggesting acupuncture may enhance facial nerve regeneration by restoring olfactory transduction-related signaling.

Downstream genes regulated by lncRNA, ENSG00000170525 (annotated as SEMA3A) and ENSG00000102870 (annotated as ROBO1), are key molecules in neural development and injury repair. The former participates in axon guidance and inhibits aberrant branching (Dent et al. 2004), while the latter, as a receptor for the SLIT family, mediates neuro-immune(Li et al. 2023) interactions. ENSG00000160233 (annotated as PLXNB1) is a receptor for axon guidance molecules like SEMA4D(Conrotto et al. 2005), regulating growth cone collapse and glial-neuronal communication (Lin et al. 2007). In PFP, axonal disruption and glial cell activation are core pathological changes post-facial nerve injury. This study found that acupuncture, via lncRNAs, can relieve miRNA-mediated post-transcriptional inhibition of these genes, potentially promoting the expression of SEMA3A, ROBO1, and PLXNB1, thereby facilitating nerve repair through their synergistic actions.

Notably, Liu et al.(Liu et al. 2025) identified the SLIT2-ROBO axis as a key therapeutic candidate target for PFP: SLIT2 can participate in neuro-immune interactions and promote nerve repair by regulating microglial inflammation, inhibitory neuron excitability, and glial-neuronal communication. This provides complementary theoretical basis and potential targets for PFP treatment from different molecular regulatory pathways.

Traditionally, the core function of the olfactory transduction pathway was confined to sensory perception (e.g., odor identification). However, recent research has expanded this boundary, confirming that core components of this pathway—olfactory receptors—hold potential as therapeutic targets for neural repair and functional recovery after central nervous system injury (Franco et al. 2025). Importantly, in PFP-related research, the target gene ENSG00000134817 (annotated as olfactory receptor OR51E2) identified here is reported for the first time. Its expression crucially promotes nerve repair, with specific mechanisms supported by related studies (Franco et al. 2025): On one hand, this gene can target the dentate gyrus (a core site of adult neurogenesis), regulating the proliferation, differentiation, and survival of neural progenitor cells, thereby promoting adult neurogenesis and supplementing functional neurons for damaged nerves (Whitman and Greer 2009). On the other hand, it can improve signal conduction and synaptic plasticity of impaired neurons by modulating neurotransmitter release efficiency, ion channel activity, and synaptic connection strength, enhancing the adaptation and repair capacity of neural networks (Avchalumov and Mandyam 2021). The JAK-STAT pathway is a core mediator of inflammation and immune responses, primarily transmitting extracellular signals like cytokines and growth factors into the nucleus to regulate gene expression. After facial nerve injury, overactivation of the JAK-STAT pathway exacerbates neuroinflammation and leads to Schwann cell dysfunction, which are crucial for nerve repair(Wei et al. 2024).

Previous studies confirmed that acupuncture can suppress JAK-STAT pathway overactivation in autoimmune diseases (O’Shea and Plenge 2012) and neuroinflammatory conditions (Wang et al. 2022). Our data extend this finding: acupuncture modulates this pathway via the ceRNA network. Specifically, the target gene ENSG00000196169 (annotated as SOCS3), a negative regulator of JAK-STAT signaling (Tamiya et al. 2011), can be upregulated through sequestration of hsa-miR-7974, thereby inhibiting excessive inflammatory responses, forming an “acupuncture-circRNA-hsa-miR-7974-SOCS3-JAK-STAT” anti-inflammatory regulatory axis.

Facial nerve damage manifests as localized focal lesions. Accordingly, the biological rationale underlying the correlations between peripheral serum transcriptional profiling and local lesion pathology, nerve injury status as well as treatment efficacy is interpreted in several aspects as follows.

According to the enrichment results of the three major GO ontologies, the four RNA molecules perform distinct biological functions and constitute a regulatory network governing neuroinflammation and tissue repair processes. At the biological process level, differentially expressed mRNAs are abundantly enriched in fundamental cellular activities, substance transport and the regulation of cellular localization. These protein-coding genes directly mediate the migration, adhesion and activation of peripheral immune cells. Following focal damage within the facial nerve canal, abundant chemotactic signals are released from lesions. Circulating immune cells infiltrate the damaged cranial facial nerve tissue under the regulation of proteins associated with intracellular localization and transmembrane transport, which acts as a central event triggering facial nerve edema and demyelination.

miRNAs are predominantly enriched in biological processes related to transcriptional and metabolic regulation. As post-transcriptional repressors, they globally suppress the expression of genes responsible for excessive immune cell activation, thereby counterbalancing the exaggerated inflammatory response induced by viral stimulation. lncRNAs mainly target RNA metabolism and the positive regulation of gene expression; inside the cell nucleus, they cooperate with transcription factors to amplify transcriptional signals of pro-inflammatory genes and exacerbate systemic immune activation. Notably, circRNAs are specifically enriched in the IL-6 signaling pathway and autophagy-related biological processes. IL-6 serves as a pivotal pro-inflammatory cytokine that drives acute local edema and inflammatory cell infiltration during facial neuritis, whereas autophagy pathways modulate Schwann cell survival and the clearance of fragmented myelin debris. These findings indicate that circRNAs act as critical mediators linking systemic inflammatory cytokine storms to local neuronal injury and repair, and explain why alterations in serum circRNA abundance can directly reflect the local inflammatory burden of the facial nerve.

Enrichment profiles of cellular components further validate the subcellular localization-specific roles of the four RNA molecules and support their hierarchical regulation of pathological progression. Protein-coding mRNAs are widely distributed in the cytoplasm and organelles to sustain basic physiological functions of immune cells. Enriched in integral components of the cell membrane, miRNAs participate in transmembrane intercellular signal transduction and mediate crosstalk between peripheral immune cells, vascular endothelial cells and glial cells. lncRNAs are mainly localized to the nucleus and nucleoplasm, executing regulatory functions at the transcriptional level. circRNAs are enriched in nuclear bodies and the trans-Golgi network. The Golgi apparatus governs the processing and secretion of inflammatory cytokines and chemokines; circRNAs aggravate local inflammatory injury of the facial nerve by modulating Golgi function to promote the release of injury mediators such as IL-6 and chemokines into peripheral blood, providing subcellular evidence for circulating circRNA levels to mirror the inflammatory severity of local lesions.

Differential enrichment at the molecular function level reveals the core functional modes of the four RNA subtypes. mRNAs provide enzymatic and molecular binding prerequisites for immune activation and cellular migration via broad nucleic acid binding, ATP binding and catalytic activities. miRNAs modulate the transcriptional efficiency of target genes through sequence-specific DNA binding and transcription factor activities. lncRNAs exert exclusive RNA-binding capacity and act as molecular sponges to sequester miRNAs and relieve the transcriptional repression of target genes. circRNAs are enriched in ribosome binding and histone methyltransferase activity, enabling dual transcriptional and post-transcriptional regulation of inflammation- and autophagy-related genes. Collectively, the four RNA molecules function complementarily to construct a peripheral blood regulatory network that collaboratively participates in local pathological injury of the facial nerve.

KEGG pathway enrichment analysis further identifies the core pathogenic triggers and injury mechanisms underlying facial neuritis in this study. Pathways enriched by the four RNA species are mostly categorized into human disease pathways with molecule-specific enrichment signatures, which is highly consistent with the viral-induced and autoimmune-mediated pathogenesis of idiopathic facial neuritis. mRNAs are markedly enriched in pathways linked to neurodegenerative disorders (Huntington’s disease), metabolically driven inflammation (non-alcoholic fatty liver disease) and thermogenesis pathways, implying that peripheral metabolic disturbance and progressive neuronal degeneration jointly contribute to facial nerve lesions. Both miRNAs and lncRNAs are primarily enriched in the herpes simplex virus type 1 (HSV-1) infection pathway, the most prevalent pathogen causing clinical idiopathic facial neuritis. Viral reactivation induces systemic lymphocyte activation and abnormal apoptotic signaling; the subsequent recruitment of circulating immune cells into the facial nerve canal eventually leads to demyelinating damage. This enrichment result directly establishes a correlative relationship between peripheral transcriptional perturbations and local virus-mediated inflammation of the facial nerve. circRNAs are specifically enriched in cancer-associated miRNA pathways and porphyrin metabolism pathways. Existing studies have demonstrated that disrupted porphyrin metabolism intensifies oxidative stress damage and accelerates Schwann cell apoptosis. Meanwhile, cancer-related miRNA pathways are extensively involved in the regulation of inflammation and autophagy, which echoes the IL-6 and autophagy pathways identified via GO enrichment for circRNAs.

Integrated analysis of hierarchical GO and KEGG enrichment patterns demonstrates that although facial nerve lesions are confined to partial neural tissue, core pathological events including viral infection, pro-inflammatory cytokine release, immune cell activation and neuronal injury and repair will reshape the expression profiles of circulating mRNAs and diverse non-coding RNAs throughout the systemic circulation. Accordingly, dynamic changes in the peripheral blood transcriptome can objectively reflect therapeutic responses characterized by local inflammatory resolution and nerve repair in facial neuritis. This study supplies comprehensive, multi-layered molecular biological evidence supporting the application of serum transcriptomic biomarkers for evaluating disease severity and therapeutic efficacy in patients with facial neuritis.

### Limitations

This study has limitations. Suboptimal data quality from some samples, with miRNA sequence alignment rates below the preset 80% threshold in a few cases, despite correction by removing extreme outliers and optimizing alignment parameters, might reduce quantification accuracy for some low-abundance miRNAs and slightly impact the precision of target prediction and pathway enrichment analysis. The sample size and coverage of clinical characteristics were limited, potentially restricting the generalizability of the findings. Only peripheral serum specimens were obtained in the current study, whereas matched transcriptomic profiling data from lesional facial nerve tissues were lacking. Hence, a direct comparison of RNA expression signatures between local lesional tissues and peripheral circulation could not be accomplished.

### Future Directions

Based on these limitations and the core research direction, future work should focus on: 1) Strictly optimizing experimental procedures to ensure subsequent sample data quality; expanding sample size, incorporating patient cohorts with varying ages, disease courses, palsy subtypes, and comorbidities; conducting stratified analyses to clarify clinical heterogeneity related to core ceRNA axes and pathway regulation, enhancing result generalizability. 2)Subsequent studies will collect perilesional facial nerve tissues to implement multi-omics orthogonal validation. Meanwhile, an external clinical cohort will be established to verify the diagnostic value and therapeutic monitoring efficacy of serum transcriptional biomarkers, thereby strengthening the evidence supporting the ability of peripheral blood indicators to mirror local neural pathological conditions. 3)Further validating mechanisms using proteomics and metabolomics to identify downstream effector proteins of the Olfactory transduction and JAK-STAT pathways, completing the “ceRNA axis-pathway-effector protein” regulatory cascade. 4) Deeply exploring clinical value: developing targeted drugs based on core targets; conducting animal experiments and clinical pilot studies on combined “acupuncture + targeted drug” therapy to explore new strategies for improving efficacy in refractory facial paralysis.

## Data Availability

No datasets were generated or analysed during the current study.
